# Status-impact assessment: is accuracy linked with status motivations?

**DOI:** 10.1017/ehs.2023.12

**Published:** 2023-05-15

**Authors:** Patrick K. Durkee, Aaron W. Lukaszewski, David M. Buss

**Affiliations:** 1 Department of Psychology, California State University, Fresno, California, USA; 2 Department of Psychology, The University of Texas at Austin, Austin, Texas, USA; 3 Institute for Advanced Study in Toulouse, Toulouse, France; 4 Department of Psychology, California State University, Fullerton, California, USA

**Keywords:** status, personality, motivation, individual differences, accuracy

## Abstract

Status hierarchies are ubiquitous across cultures and have been over deep time. Position in hierarchies shows important links with fitness outcomes. Consequently, humans should possess psychological adaptations for navigating the adaptive challenges posed by living in hierarchically organised groups. One hypothesised adaptation functions to assess, track, and store the status impacts of different acts, characteristics and events in order to guide hierarchy navigation. Although this status-impact assessment system is expected to be universal, there are several ways in which differences in assessment accuracy could arise. This variation may link to broader individual difference constructs. In a preregistered study with samples from India (*N* = 815) and the USA (*N* = 822), we sought to examine how individual differences in the accuracy of status-impact assessments covary with status motivations and personality. In both countries, greater overall status-impact assessment accuracy was associated with higher status motivations, as well as higher standing on two broad personality constructs: Honesty–Humility and Conscientiousness. These findings help map broad personality constructs onto variation in the functioning of specific cognitive mechanisms and contribute to an evolutionary understanding of individual differences.

## Graphical abstract

**Figure:**
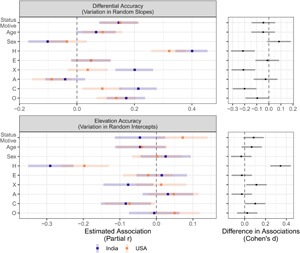

**Social media summary:** We investigate how individual variation in status impact assessment relates to motivation and personality.

Status hierarchies are ubiquitous features of group-living species (Anderson et al., 2015; Brown, 1991) with important links to many components of fitness ranging from mating opportunities to better health outcomes for children (Alami et al., 2020; Cowlishaw & Dunbar, 1991; Jaeggi et al., 2021; Majolo et al., 2012; Patton, 2000; Redhead & von Rueden, 2021; von Rueden & Jaeggi, 2016; von Rueden et al., 2019; von Rueden et al., 2011). Status pursuit and other aspects of hierarchy navigation would have been important adaptive problems for our ancestors, and we should expect that humans possess psychological adaptations that aid hierarchy navigation (Kyl-Heku & Buss, 1996; Tooby & Cosmides, 1990). Individual variation in the efficacy of adaptations involved in hierarchy navigation may be linked to broader individual difference constructs (e.g. personality traits), but little is known about such links. In the current investigation, we aim to examine how variation in the functioning and outputs of one such adaptation – the status-impact assessment system – relates to other individual difference constructs, such as motivation and personality.

## Status-impact assessment

Across cultures, an individual's status within human groups can be affected by countless acts, strategies, characteristics and events that are relevant to interpersonal valuations within one's group (Anderson et al., 2001; Buss et al., 2020; DesJardins et al., 2015; Durkee et al., 2020; Henrich & Gil-White, 2001; von Rueden et al., 2008). To successfully navigate hierarchies, individuals must coordinate behaviours and advertise characteristics that would increase their status while simultaneously avoiding those that would harm their status (Durkee et al., 2019; Sznycer, 2019). People also need to be able to estimate the relative status of self and others with probabilistic accuracy (Desmichel & Rucker, 2022; Durkee, 2021; Yu & Kilduff, 2020). Thus, the human mind may contain mechanisms that function to assess and store the expected status impacts of different acts, events and characteristics – just as there are evolved mechanisms designed to assess relative formidability (Durkee et al., 2018; Sell et al., 2009) and attractiveness (Andrews et al., 2017; Sell et al., 2017), likelihood of pathogen risk (Tybur & Lieberman, 2016), others’ relationship quality (Bryant et al., 2016, 2018), personality differences (Buss, 2011; Lukaszewski et al., 2020) and many more dimensions of fitness-relevant information. These status-impact assessments could be referenced by other mechanisms, such as emotions, to guide tactics and behavioural strategies that facilitate hierarchy navigation (Durkee, 2021; Kyl-Heku & Buss, 1996; Lund et al., 2007).

How might estimates of status impacts be instantiated within the mind? Like other internal regulatory variables (cf. Tooby et al., 2008), internal estimates of status impacts could be informed by evolved priors based on ancestrally recurrent links between personal characteristics and status. For example, generating benefits for one's ingroup is likely to have reliably increased status in the ancestral past, whereas failing to generate benefits or inflicting costs on ones’ ingroup probably lowered it (Durkee et al., 2020). If throughout our evolutionary history some characteristics or acts would have reliably generated benefits for others within one's ingroup (e.g. being a good hunter, being cooperative, coordinating collective actions) or harmed them (e.g. being selfish, being lazy, violating social contracts), cognitive representations of probabilistic status impacts of these acts or characteristics may have been innately coded within the mind over deep evolutionary time. These status-impact estimates should also be expected to be updated across development to adapt to predictable variations in the local cultural, interpersonal and physical ecology. For example, hunting ability may be less important than warriorship in one group but more important in another; intelligence may be more important than attractiveness in one group but less important in another; and these relative rankings may change over time as one enters different social niches or life history stages. Additionally, many status criteria may be evolutionarily novel, for instance, skill in computer coding or owning a nice car. Such novel inputs would need to be integrated within existing templates of benefit generation and cost infliction affordances or nested under existing status-criteria categories (e.g. ‘computer coding’ nested within ‘possess useful skills’, and ‘nice car’ within ‘possess valuable resources’).

Extant research provides evidence for the existence of a status-impact assessment system, which likely comprises a bundle of partially distinct but interconnected mechanisms – and which could itself be component of a broader status management system. For instance, people within and across cultures exhibit strong agreement about the relative impacts of different personal characteristics on interpersonal value and social status (Durkee et al., 2019; Sznycer et al. 2016, 2017, 2018a, b). Moreover, these estimates appear to be involved in the activation of a wide range of social emotions designed to coordinate behaviours that aid in hierarchy navigation, such as pride and shame (Durkee et al., 2019; Sznycer & Cohen, 2021a, b; Sznycer & Lukaszewski, 2019; Witkower et al., 2020). Together, these findings provide evidence of a status-impact assessment system within the human mind that relies on a reference of estimates of the status impacts of different acts, characteristics and events to guide hierarchy navigation. The apparent existence of a universal status-impact assessment system does not, however, preclude the possibility of predictable and important individual variation in such an adaptation.

## Individual differences in status-impact assessments

There are at least five non-exclusive explanations for individual variation in a status-impact assessment system, even if the computational architecture of the adaptation is universal (cf. Buss & Penke, 2015). First, *de novo* mutations could create individual variation in prior estimates of status impacts or in the system's ability to accurately update and store estimates based on environmental inputs. Second, balancing selection could, in principle, maintain different levels of assessment accuracy within a population under certain circumstances. Third, developmental insults that affect the normative development of the status-impact assessment system or any other mechanisms it receives input from could affect manifest accuracy. Fourth, differences in the inputs an individual receives across development could result in different assessments of status impacts that could be mismatched to their current social environment. Fifth, variation could result from individual differences in resources devoted to status-impact assessments relative to other adaptive problems.

Although the desire for status appears to be a fundamental human motive (Anderson et al., 2015), there are many other fundamental goals to which humans must allocate limited energy budgets (Kenrick et al., 2010; Ko et al., 2020). Energy and time allocated to these other fundamental goals cannot be allocated to status pursuit. For example, cultivating skills or traits that could enhance one's status may require sacrificing energy or time that – in the short-term – could be devoted to opportunities for food, courtship, kin investment, or offspring care. Additionally, some fundamental goals, such as self-protection, may directly conflict with the development or demonstration of some status-increasing characteristics, such as bravery in the face of danger or winning agonistic contests. Although fulfilling some fundamental motives (e.g. finding a mate, investing in relationships) may ultimately further status pursuit and energy allocated to status pursuit can jointly further other motives when successful, a tradeoff exists between resources devoted to status pursuit and to other motives given finite time and energy budgets (Buss & Penke, 2015). Motivations may help to solve this superordinate adaptive problem by acting as control systems or weights that regulate the distribution of finite resources among conflicting goals (Del Giudice, 2022). Specifically, higher status motivations may serve to direct relatively more energetic resources towards status-relevant goals and information when fundamental goals are in conflict.

Given the theorised role of motivations in regulating resource distribution across diverse adaptive problems, there are multiple, non-exclusive potential causes^1^ of an association between status-impact assessment accuracy and status motivations. If status pursuit is weighted as lower or higher in priority than other goals, this may lead to between- and within-person variation in energy allocated to status pursuit mechanisms. For example, low status motivations may be associated with reduced attention to status-relevant information and less energy allocated towards updating estimates of status impacts, resulting in less accurate status-impact assessments. Alternatively, variation in status-impact accuracy may itself drive differences in status motivations. For instance, relatively accurate status-impact assessments could result in successful status pursuit attempts, and this positive feedback could up-regulate motivations to pursue status over time to adaptively allocate energetic resources among competing goals. Of course, the causal pathway may be much more complicated, with feedback loops and additional variables that regulate both status-assessment accuracy and status motivations.

In support of the rationale for links between motivations and the accuracy of status-impact assessment mechanisms, some indirect evidence suggests that status motivations are indeed associated with the functioning of other, closely related hierarchy navigation mechanisms. An ancillary finding of Yu and Kilduff's (2020) study examining variation in individuals’ perceptions of their group's hierarchical structure was that people whose mental representation of the hierarchy was closer to the average rankings of all group members (i.e. more accurate perceptions of the hierarchy) tended to have higher scores on a measure of status motivation than did individuals with less accurate perceptions of the group hierarchy (*r* = 0.12, *p* = 0.02). Given that these perceptions of the group hierarchy result from mechanisms to assess hierarchical structure and rankings of group members, this finding provides some initial evidence that the functioning of hierarchy navigation mechanisms does vary with status motivations. The mechanisms that assess the status impacts of different acts, characteristics and events are unlikely to be isomorphic with the mechanisms designed to assess the existing hierarchical rankings of one's group; however, they may depend on similar information-processing structures and may both be linked to differences in status motivations. Although Yu and Kilduff's (2020) finding is suggestive that the accuracy of the status-impact assessment system could be related to individual differences in status motivations, there have been no direct tests of this relationship.

## The current research

Given the theoretical rationale and indirect evidence detailed above, we developed an empirical study to directly test the hypothesis that status-impact assessment accuracy will be positively associated with individual differences in status motivations. Following previous research (e.g. Anderson et al., 2006; Yu & Kilduff, 2020), we distinguish between (a) *elevation accuracy*, which describes the extent to which a person tends to over- or under-estimates status-impacts compared to others, and (b) *differential accuracy*, which describes the degree to which an individual's status-impact perceptions track the rank-ordering of their peers. Specifically, we predict that people who are more highly motivated to attain status will have more accurate assessments of the relative status impacts of different acts, characteristics and events (i.e. better differential accuracy), and will also tend to overestimate status impacts on average compared to people with comparatively lower status motivations (i.e. higher elevation accuracy).

We will also explore whether and how HEXACO personality traits are associated with status-impact assessments and status motivations in order to further an adaptationist framework for understanding the underpinnings of broad personality traits (cf. Lukaszewski et al., 2020). For example, the downstream effects of less-accurate assessments on status-seeking behaviours (e.g. avoiding status-damaging acts) could be associated with behavioural variation that leads people to be perceived as lower on Conscientiousness, Honesty–Humility or Emotionality. We focus on the HEXACO personality framework rather than the Big 5 primarily because of the additional dimension of Honesty–Humility, which on its face is likely to be relevant to status-related individual differences. Additionally, growing evidence suggests that the HEXACO framework exhibits stronger predictive power than the Big 5 (for a review, see Feher & Vernon, 2021).

Finally, some have argued that males gain more potential fitness benefits from status than do women, and that this may have selected for higher status motivation in males than females on average (e.g. Buss, 1999; Campbell, 1999). However, this this has not been adequately tested in high-powered studies. Because we will be measuring status motivations as part of our primary research question, we will also test this prediction. Relatedly, we will test whether there is a sex difference in the accuracy of status impacts assessments. If men are more status-motivated than women on average, and status motivations are associated with accuracy in assessing status impacts, then it could be predicted that men will exhibit higher differential and elevation accuracy.

To test these predictions and questions, we first conducted a pilot study in American college students (detailed in the Supplementary Information) before conducting the following registered report as a high-powered replication. We collected large samples of participants from India and the USA and employed multiple measures of status motivations to better assess the generalisability of our findings. The approved Stage 1 protocol is available on the Open Science Framework (https://osf.io/7jm2r/) and any deviations from the preregistered design are noted in the text.

## Method

### Participants

Power analyses based on the pilot study suggested that 400 men and 400 women would allow us to detect the expected small effects (*r ≈* 0.1; *d ≈* 0.2) with 80% power (for full power analysis details see the Supplementary Information). We collected over 400 men and 400 women from both India (*N* = 868) and the USA (*N =* 1056) using the Cloud Research platform to recruit relatively balanced numbers of men and women from Amazon Mechanical Turk. Participants were paid $1.00 for completing the study. Following our preregistered exclusion criteria, we removed 41 participants in the USA sample who failed the attention check and five who did not self-identify as either male or female; we excluded 231 participants from the India sample who failed the attention check, three who did not self-identify as either male or female and seven who failed an English comprehension question. The final sample sizes are *n* = 815 (411 women) in India and *n* = 822 (412 women) in the USA. The age of participants ranged from 18 to 80 in the USA (mean, *M* = 44.14, standard deviation, SD = 14.31) and from 19 to 73 in India (M = 33.61, SD = 7.65).

### Study materials and procedures

The university's Institutional Review Board approved the study and all participants provided informed consent prior to participation. Participants accessed the study via Amazon Mechanical Turk and completed the survey on Qualtrics. After providing demographic information (e.g. age, sex, ethnicity), participants completed three additional questionnaires designed to measure their (a) assessments of the status impacts of different personal characteristics, (b) status motivation and (c) standing on HEXACO personality traits. The order in which the questionnaires were presented was randomised for each participant.

#### Status impact assessments

Participants were presented with a unique random subset of 40 different acts, characteristics and events (henceforth *personal characteristics*) in a random order and asked, ‘If people thought that you [*insert random status item*], what impact do you think this would have on your status in the eyes of other people your age?’ using a seven-point scale (−3 = ‘greatly decrease your status’; 0 = ‘has not impact on your status’; +3 = ‘greatly increase your status’). The 40 personal characteristics participants were taken from a larger set of 150 personal characteristics used in previous research investigating status criteria (Buss et al., 2020; Durkee et al., 2019, 2020); the subset was created by removing similarly worded items and items that tapped the same conceptual space. Example items include ‘were physically dominated by someone’, ‘were brave in the face of danger’, ‘were a good dancer’, ‘failed to perform a group task’ and ‘had a wide range of knowledge’. We decided to present 40 items to participants because this number most efficiently balanced the goals of maximising power to reliably detect effects in the focal models while minimising participant burden (see supplemental power analysis). The full list of 150 items used in the current study is provided on the OSF.

#### Status motivations

To measure individual differences in levels of status motivations, we employed two different scales: the Need for Status Scale developed by Flynn et al. (2006) and the status subscale of the Fundamental Motives Questionnaire (Neel et al., 2016). The Flynn et al. scale is made up of eight items (e.g. ‘I want my peers to respect me and hold me in high esteem’ and ‘I enjoy having influence over other people's decision making’). Six items comprise the status subscale of the Fundamental Motives Questionnaire (e.g. ‘I do things to ensure that I don't lose the status I have’ and ‘I do not worry very much about losing status’). On both scales, participants indicated the extent to which they agreed with each item using a seven-point scale (1 = strongly disagree; 7 = strongly agree). Reliabilities were moderately high for each scale in both the USA and India (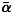
= 0.88; see Supplementary Information for more detailed reliability information).

#### HEXACO personality traits

We assessed participants standing on the HEXACO personality traits using the brief HEXACO inventory (de Vries, 2013). The scale consists of 24 items, with four items for each HEXACO personality dimension: Honesty–Humility (e.g. ‘I would like to know how to make lots of money in a dishonest manner’), Emotionality (e.g. ‘I am seldom cheerful’), Extraversion (‘I easily approach strangers’), Agreeableness (‘I often express criticism’), Conscientiousness (‘I work very precisely’), Openness (‘I like people with strange ideas’). As is typical of the brief HEXACO inventory owing to its small number of items per trait and broad coverage of trait space, the reliability coefficients were generally low in both countries (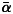
= 0.38; see Supplementary Information for more detailed reliability information). Previous validation studies, however, show that the brief HEXACO inventory exhibits strong convergent correlations with full-length scales, test–retest reliability and self–other agreement (de Vries, 2013), even though the alpha reliabilities are low.

#### Statistical software

We conducted all data cleaning, analysis, and visualisation in R (R Core Team, 2020). The packages used are *tidyverse* (Wickham et al., 2019) for data carpentry, *lme4* (Bates et al., 2018) and *lmerTest* (Kuznetsova et al., 2017) for multilevel modelling, *psych* (Revelle, 2020) and *ggstatplot* (Patil, 2018) for correlations. All data and code to reproduce the reported results are provided on the OSF (https://osf.io/7jm2r/).

#### Analytic procedure

Typically, elevation accuracy would be calculated as the mean of the differences between a person's assessments and their peer's assessments across targets or items, and differential accuracy would be calculated as the correlation between each person's assessments and the average assessments of their peers for each target or item. These estimates of differential and elevation accuracy would then be carried forward to other analyses where they are modelled as predictors or outcomes of other individual differences. However, Biesanz (2010) noted that this two-step approach ‘neither incorporates measurement error into the analysis nor provides estimates of the extent to which there actually are individual differences’ (p. 858) and suggested to use random effects models, which can efficiently model such associations in one step and estimate the amount of variability in accuracy.

To model these parameters, we used *lmer* in the lme4 package to construct a multilevel model where we regressed each participants’ self-ratings of the impact that the randomly selected set of 40 status items on (a) the average peer-rating of each item (i.e. a column containing the mean for each status item based on the ratings of every other participant not including the current participant), (b) the focal individual difference characteristics (i.e. age, sex, status motivations, HEXACO traits) and (c) the interactions between individual difference characteristics and averaged peer-ratings. We specified random intercepts and slopes of peer-ratings for participants, which respectively correspond to an individuals estimated elevation accuracy and differential accuracy. The individual difference characteristics were not included as random slopes because there is no within-cluster variability in these between-subject variables.

Because we want to estimate differential accuracy, we standardised self-assessed status and peer-assessed status within participants. Without this within-cluster standardisation, steeper slopes in the regression would not necessarily correspond to accuracy because slopes that exceed 1 are moving away from perfect accuracy.^2^ Additionally, this within-person standardisation is more appropriate for our research question examining within-person status-assessment processes and yields more accurate model estimates than global standardisation (Wang et al., 2019). We did not centre the self-impact ratings within the cluster because this would make every person's intercept zero and preclude estimating elevation accuracy; additionally, we standardised without centring the peer-assessed status impacts so that the random intercepts correspond to a person's self-assessed status impact when the peer-assessed impact is zero. To aid in interpretation of the associations with the individual difference characteristics, we grand-mean centred and standardised the HEXACO trait scores, grand-mean centred age (because one unit of age is a meaningful unit), and effect coded sex (−1 = male, 1 = female).

In our model, the main effect of peer-assessed status corresponds to the estimated accuracy across the population or agreement between the average person and their peers on the status impacts of different characteristics. The associations between individual difference characteristics (i.e. status motivation, HEXACO traits, age, and sex) and elevation accuracy correspond to the main effects of the individual difference characteristics. The associations between differential accuracy and the individual difference characteristics are captured by the interaction terms with peer-assessed status impacts. To aid in interpreting the magnitude of the effects, we used the *t_to_r* function in the *effectsize* package (Ben-Shachar et al., 2020) to compute partial correlation estimates or Cohen's *d*, as well as 95% CI for each, from the *t*-statistics and degrees of freedom provided by the multilevel model.

## Results

**Figure 1.**
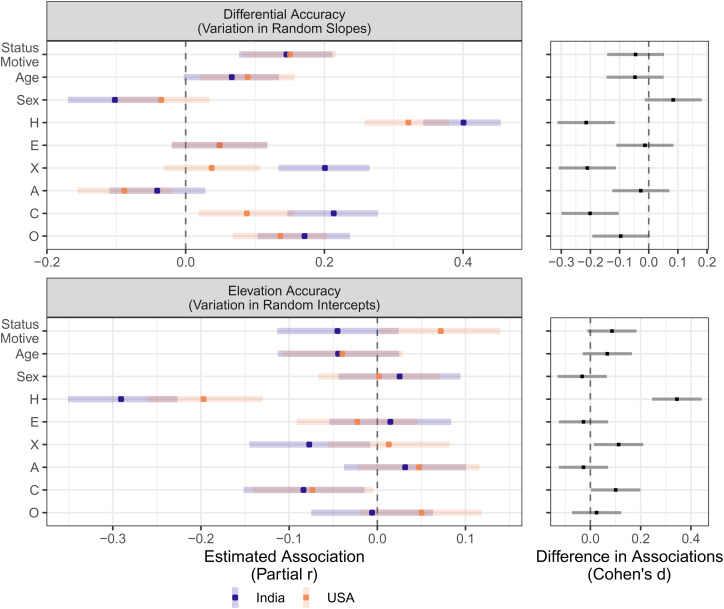
Associations between individual difference characteristics and accuracy indices for participants in the USA (light orange) and India (dark purple). The plots on the left show model-estimated associations (converted to partial correlations) and 95% confidence interval (CI) bands for the in each country. The plots on the right show exploratory contrast tests of the magnitude of the difference in the estimated associations between the two countries (converted to Cohen's *d*) and 95% CI for the difference. H, Honesty–Humility; E, emotionality; X, extraversion; A, agreeableness; C, conscientiousness; O, openness. Status motivation and the HEXACO traits are grand-mean centred and standardised, Sex is an effect coded variable where −1 = male and 1 = female. Age is grand mean centred only.

The results of our focal model examining whether status motivations predict the accuracy components across the two countries when controlling for other individual difference characteristics are summarised in Figure 1. The points depict standardised estimates computed from the model-estimated *t*-statistics and degrees of freedom, and the bands depict the 95% confidence intervals. Note that the *elevation accuracy* facet of the plot depicts the main effects of the individual difference characteristics on self-assessed status impacts (i.e. does the characteristic predict intercept variability?), and the *differential accuracy* facet depicts the effects of the interactions between each individual difference characteristic and the peer-assessed impacts on self-assessed status impacts (i.e. does a characteristic predict slope variability?). We also conducted analyses testing whether the effects differed statistically between countries, and these differences are presented in the facets alongside the focal associations in Figure 1. Although not preregistered, we explicitly tested whether the observed associations differed depending on the status motive measure that we used; we found no statistical difference between the models using the two different scales and the estimates are essentially identical, so for efficiency of presentation we only present the results based on the Neel et al. (2016) status motive measure here in the main text. In the Supplementary Information, we demonstrate that essentially identical results using the other status motive scale, and we present detailed result tables with raw estimates for models with and without controls.

There were small statistically significant interactions between status motivations and peer-assessed status impacts in both the USA sample and the India sample, suggesting that people in both countries who had higher status motivations tended towards higher differential accuracy (i.e. steeper random slopes) than those who scored lower on status motivations. The main effect for status motivation was positive and barely statistically significant in the USA sample but negative and not statistically significant in the India sample, offering next to no evidence for a reliable relationship between status motivations and elevation accuracy (i.e. random intercepts).

Differential accuracy (i.e. random participant slopes) was further predicted by several other individual difference characteristics. Age was positively associated with differential accuracy in the USA but not India, although this difference between the associations was not itself statistically significant. Self-reporting sex as female was associated with lower differential accuracy in India but not the USA, but the difference in these associations was not itself statistically significant. Honesty–Humility was reliably positively associated with differential accuracy in both the USA and India, and the association was statistically stronger in India. Extraversion was reliably positively associated with differential accuracy in India but not the USA, and this difference was statistically significant. Agreeableness was reliably negatively associated with differential accuracy in the USA but not India, although this relationship was not itself statistically significant. Conscientiousness was reliably positively associated with differential accuracy in both the USA and India, and this association was stronger in India. Finally, Openness was reliably positively associated with differential accuracy in both countries.

**Figure 2.**
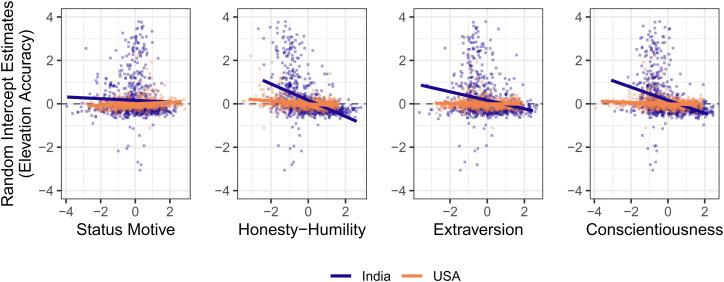
Scatterplots of associations between participant intercepts (i.e. elevation accuracy) and selected individual difference constructs in India (dark purple) and the USA (light orange). Importantly, the statistical tests of these trends reported in the text were based on associations with participants’ latent intercepts, not the extracted estimates of their intercepts depicted here which are used only to aid interpretation.

Elevation accuracy (i.e. random participant intercepts) was not statistically significantly associated with age, sex, Emotionality, Agreeableness or Openness in either country. There was a statistically significant difference in the extent to which Extraversion predicted elevation accuracy across countries: they are negatively associated in India but not statistically associated in the USA. Honesty–Humility and Conscientiousness were negatively associated with elevation accuracy in both countries, and both associations were statistically stronger in India. Because participants with perfect elevation accuracy would have an estimated intercept of zero, the population estimates themselves do not reveal whether people scoring high on an individual difference characteristic tend to over- or underestimate, so interpretation of these statistically significant elevation associations is aided by Figure 2. Examination of the scatterplots suggests that the negative associations between elevation accuracy and broad personality traits are driven by people lower in Honesty–Humility, Extraversion and Conscientiousness who tend to overestimate status impacts, while those higher on these personality traits tend to underestimate status impacts.

The fixed-effect estimates also show that participants’ self-assessments of the status impacts are very strongly positively associated with peer-assessments in both India (*r = 0*.82 [0.80, 0.83]) and the USA (*r = 0*.96 [0.96, 0.96]); the difference in slopes between the countries was statistically significant (*d* = 0.73 [0.63, 0.83]). The estimated population-level intercept in was not statistically different from zero in the USA (*b =* 0 [−0.07, 0.07] but reliably positive in India (*b* = 0.18 [0.12, 0.25]) – and this difference was statistically significant (*d* = 0.26 [0.16, 0.36]) – suggesting that the average participant in the USA would perceive a status impact of zero when peers deem it to be zero, whereas Indian participants may tend to perceive non-zero status impacts even when their peers see none. Examination of the variance components showed there was qualitatively less variation in the random effects parameters across participants in the USA (slope *σ* = 0.18; intercept *σ* = 0.23) than participants in India (slope *σ* = 0.35; intercept *σ* = 1.13). Additionally, the variance components reveal that latent participant slopes and intercepts are moderately negatively correlated in both the USA (*r =* −0.33) and India (*r =* −0.55), suggesting that they are capturing at least partially distinct aspects of status-impact assessment accuracy as we have measured it.

**Figure 3.**
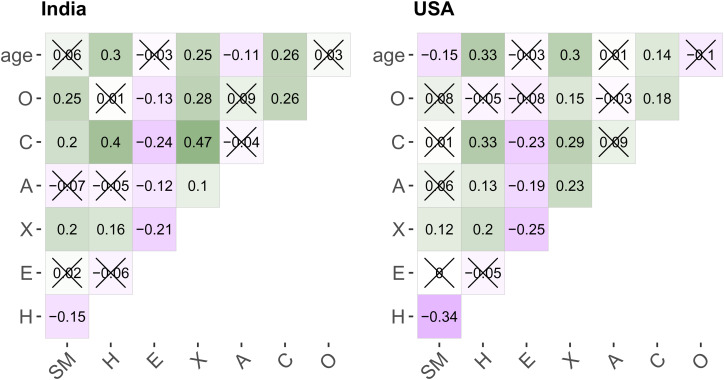
Correlation matrices depicting the magnitude, direction, and statistical significance of the country-specific correlations between the individual difference variables collected in the present study. All *p*-values are adjusted for multiple tests using Holm's method. Crossed-out correlations are not statistically significant. SM, Status motive; H, Honesty–Humility; E, emotionality; X, extraversion; A, agreeableness; C, conscientiousness; O, openness.

Figure 3 shows the zero-order correlations between the individual difference constructs we assessed in the current study. The pairwise correlations were largely directionally consistent in both the USA and India.

Finally, to investigate whether there may be a sex difference in overall status motivations, we conducted a Welch two-sample *t*-test in each country. There was not a statistically significant difference between women's (*M* = −0.04, SD = 1.00) and men's (*M* = 0.04, SD = 1.01) status motivation in the USA (*d =* 0.09 [−0.05, 0.22]); nor between women's (*M* = 0.04, SD = 0.94) and men's (*M* = −0.04, SD = 1.06) status motivation in India (*d* = 0.08 [−0.06, 0.22]).

## Discussion

The primary aim of this registered report was to test whether status-impact assessment accuracy is associated with status motivations. We predicted that people who are more motivated to attain status would tend to (a) overestimate status impacts on average (i.e. have higher elevation accuracy) and (b) be more accurate in assessing the relative status impacts of different personal characteristics (i.e. have higher differential accuracy). We tested the predictions using samples of participants from the USA and India, as well as two different measures of status motivation, to examine the generalisability of the relationship. We did not find reliable evidence for a positive association between status motivation and *elevation accuracy*. We did, however, find evidence for a reliably small positive association between status motivation and *differential accuracy* that was generalisable across both countries and measures.

Taken together, the pattern of results suggests that people who are more motivated to attain status tend to be slightly more accurate overall in assessing relative status impacts of acts, characteristics and events. Future research may be able to tease apart the directional effects. For example, studies could manipulate participants’ ability to learn status criteria in novel social environments to see if this increases their status motivations, or alter the cost–benefit ratio of striving for status to see whether this lowers participants’ accuracy in assessing status criteria in novel social environments.

Our study also investigated associations between status-impact assessment accuracy and broader personality constructs. Within the HEXACO taxonomy, we found that Emotionality, Extraversion, Agreeableness and Openness were only very weakly or inconsistently related to the indices of status-impact assessment accuracy. Conscientiousness and Honesty–Humility, however, were each consistently related to both accuracy indices in both countries. We note that the reliabilities of the Agreeableness and Emotionality subscales were generally poor in the India sample (see Supplementary Information), suggesting that these traits may not be adequately represented in our model; this could be problematic if these traits are confounded with other personality traits (Westfall & Yarkoni, 2016). Given the generally low overlap among HEXACO trait constructs across studies, it seems unlikely to us that the reliability issues should be expected to explain away the associations that we did find consistently across samples. Thus, while we remain confident that status-impact assessment is linked to Honesty–Humility and, to a lesser extent, Conscientiousness, we would not rule out the possibility that more focused measures of Agreeableness or Emotionality may reveal links to status-impact assessment as well.

Personality frameworks offer different interpretations of associations between status-assessment accuracy and broader personality constructs, depending on the ontological status they ascribe to traits. Under core trait perspectives (e.g. McCrae & Costa, 2008), the trait Conscientiousness could lead to paying more attention to status-relevant information (or to survey questions in general) and thus greater accuracy. Being low on the trait Honesty–Humility, which overlaps substantially with Machiavellian and psychopathic tendencies (Lee & Ashton, 2005), may lead people to perceive different tactics as being useful for getting status than their peers, making them less accurate. Alternatively, people low in Honesty–Humility may disagree with most others about how many behaviours should impact one's status, but possess accurate knowledge of alternative tactics for gaining status that are socially disvalued. Under emergentist perspectives (e.g. Mischel & Shoda, 1995; Uher, 2013), people whose status-impact assessments are more accurate may behave in ways that lead them to be perceived as Conscientious by others (and themselves). Likewise, relatively inaccurate status-impact assessment may increase the frequency or probability of behaviour labelled by our folk psychologies as greedy, pretentious, entitled, immodest and other lexical terms ultimately summarised by the Honesty–Humility construct. While more research is necessary to adjudicate among these interpretations, the emergentist framework arguably invokes fewer causal entities and is therefore more parsimonious (Wood et al., 2015). Under this perspective, our findings contribute to identifying the social–cognitive underpinnings of broad descriptive personality constructs (Lukaszewski et al., 2020; Mõttus et al., 2020).

Contra to hypotheses in the literature that status striving would be higher in men than women because men can more easily convert status into increased reproductive success (e.g. Buss, 1999; Campbell, 1999), we found no evidence of a sex difference in status motivations in India or the USA. We did find that women had slightly lower differential accuracy in the Indian sample, but we did not find a similar sex difference in the USA sample, nor were there any differences in elevation accuracy (i.e. tendency to over- or underweight status impacts). The lack of a sex difference is perhaps not surprising. After all, women across diverse societies could also efficiently convert status into increased reproductive success, for example, via increased offspring health (Alami et al., 2020). Sex differences in status-relevant aspects of psychology may instead be found in the specific strategies and tactics used to navigate hierarchies – to the extent that these reflect differences in fitness costs and benefits of different behaviours for ancestral males and females. In sum, there does not appear to be good evidence for sex differences in the status-relevant motives and perceptions captured by our study; however, more theoretical and empirical research is needed to explore possible sex differences in the nuances of status motives.

Our study also featured an underused approach to modelling assessment accuracy. Much previous research has essentially treated elevation and differential accuracy as observed variables without error (e.g. Anderson et al., 2006). Analyses using such estimates as predictors or outcome variables will underestimate the uncertainty around results, contributing to a higher false positive rate. Our analysis approach borrows from methods in the person perception literature (e.g. Biesanz & Wallace, 2020) to address this issue by modelling accuracy as latent intercepts and slopes within a multilevel model which carries forward the uncertainty around these accuracy components into estimations of their associations with other individual difference variables. We hope our study highlights how this modelling strategy could be fruitfully applied to more effectively model accuracy in many domains.

Overall, our findings suggest that small differences in the accuracy with which people assess the status impacts of acts, characteristics and events are linked to motivations for status and broader personality constructs in at least two culturally dissimilar societies. Although the links are relatively weak, status assessment is just one small piece of a complex cognitive architecture composed of many psychological mechanisms that are hypothesised to coordinate responses to myriad adaptive challenges. Variation in any given component of this psychological toolkit may only explain a small portion of broader differences. Just as population genetics research has demonstrated that complex traits are highly polygenic with many genes having small effects, perhaps we should likewise expect broad dimensions of individual differences to be highly *polymechansistic* – made up of many small differences in many psychological mechanisms. Future research systematically examining the overlap among individual and cultural variation in the functioning of specific cognitive mechanisms, motivations and personality traits can contribute to a more fine-grained understanding human differences and similarities.

## Footnotes

1 We note that the current correlational study is not intended to assess causal pathways and is not designed as such.

2 We thank the handling editor for pointing out this issue. In the Supplementary Information, we show that the predicted slopes from this within-cluster standardization do not exceed 1. We also show that the slopes and intercepts estimated from the multilevel model are respectively strongly correlated with the typical ways of calculating differential and elevation accuracy, suggesting that we are modeling accuracy as intended.

## Supplementary material

For supplementary material accompanying this paper visit https://doi.org/10.1017/ehs.2023.12 click here to view supplementary material

## Research transparency and reproducibility

All data, materials, and code for the Stage 1 and Stage 2 registered report submission are available on the OSF: https://osf.io/7jm2r/
