# Supplementary material for: Status-impact assessment: is accuracy linked with status motivations?
Source: Evol Hum Sci. 2023 May 15;5:e17. doi: 10.1017/ehs.2023.12 (PMC10426072; doi:10.1017/ehs.2023.12)
Supplement: Supplementary file 1 [file S2513843X23000129sup001.pdf]

Supplement

Table of Contents

Stage 1 Submission Pilot Study 2

Registered Report Power Analysis 6

Comparing the typical way of computing accuracy components to the multilevel model estimation of these components... 8

Scale information 9

Same results with another measure of status motivation 10

Detailed model results tables 11

Supplemental References 14

Stage 1 Submission Pilot Study

Prior to submitting the registered report, we conducted a pilot study to assess our planned survey methods and statistical approach, as well to get a sense of the effects we might expect to plan sample sizes for the registered report. Below we report the methods and results of the pilot study that were submitted as part of the Stage 1 submission.

Participants

We conducted a pilot study with 290 participants at a large university in Texas who participated in return for partial fulfillment of a requirement for course credit. We removed data from 49 participants who did not pass two attention checks that were embedded in the survey and one person who did not self-identify as either male or female. The final sample includes data from 240 participants (137 women and 103 men) aged 18-25 (M = 19.64, SD = 1.26). In this final sample, the breakdown of self-reported ethnicities was 31.25% Asian, 3.33% Black, 32.50% Latinx, 28.75% White, and 4.17% Other.

Study Materials and Procedures

The methods were identical to those reported in the main text, except only one measure of status motivation was used and all 150 status items were presented to all participants, rather than 40.

The university's IRB approved the study and all participants provided informed consent prior to participation. Participants accessed the study via the university SONA system and completed the survey on Qualtrics. After providing demographic information (e.g., age, sex, ethnicity), participants completed three additional questionnaires designed to measure their (1) assessments of the status impacts of different personal characteristics, (2) status motivation, and (3) standing on HEXACO personality traits. The order in which the three questionnaires were presented was randomized for each participant.

Status impact assessments. Participants were presented with 150 different acts, characteristics, and events (henceforth *personal characteristics*) in a random order and asked, "If people thought that you [*insert random status item*], what impact do you think this would have on your status in the eyes of other people your age?" using a 7-point scale (-3 = "greatly decrease your status"; 0 = "has not impact on your status"; +3 = "greatly increase your status"). The 150 personal characteristics participants were taken from a larger set of 240 personal characteristics used in previous research investigating status criteria (Buss et al., 2020; Durkee et al., 2019; Durkee et al., 2020); the subset was created by removing similarly worded items and items that tapped the same conceptual space. Example items include, "were physically dominated by someone", "were brave in the face of danger", "were a good dancer", "failed to perform a group task", "had a wide range of knowledge". The full list of 150 items used in the current study is provided on the OSF (https://osf.io/7jm2r/?view_only=d81be758d59e4eda80b4428a95fb7c27).

Status motivations. To measure individual differences in levels of status motivations, participants then completed the "Need for Status Scale" developed by Flynn et al. (2006). The scale is made up of eight items (e.g., "I want my peers to respect me and hold me in high esteem", "I enjoy having influence over other people's decision making"). Participants indicated the extent to which they agreed with each item using a 7-point scale (1 = strongly disagree; 7 = "strongly agree). The scale reliability was high (α = .83).

HEXACO personality traits. We assessed participants standing on the HEXACO personality traits using the brief HEXACO inventory (de Vries, 2013). The scale consists of 24 items, with four items for each HEXACO personality dimension: Honesty-Humility (e.g., "I would like to know how to make lots of money in a dishonest manner"; α = .41), Emotionality (e.g., "I am seldom cheerful"; α = .39), Extraversion ("I easily approach strangers"; α = .59), Agreeableness ("I often express criticism"; α = .49), Conscientiousness ("I work very precisely"; α = .49), Openness ("I like people with strange ideas"; α =

.42). Relatively low internal reliability is typical of the brief HEXACO inventory due to its small number of items per trait and broad coverage of trait space; however previous validation studies show that it exhibits strong convergent correlations with full-length scales, test-retest reliability, and self-other agreement (de Vries, 2013).

Analytic Procedure. *The analytic procedures were identical to those in the preregistered study and reported in the main text.*

Results

The results of our main model examining whether status motivations predict the accuracy components when controlling for other individual difference characteristics are summarized in Figure 1. The points depict partial correlations computed from the model-estimated *t*-statistics and degrees of freedom, and the bands depict the 95% confidence intervals. Note that the *elevation accuracy* facet of the plot depicts the main effects of the individual difference characteristics on self-assessed status impacts (i.e., does the characteristic predict intercept variability?), and the *differential accuracy* facet depicts the effects of the interactions between each individual difference characteristic and the peer-assessed impacts on self-assessed status impacts (i.e., does a characteristic predict slope variability?).

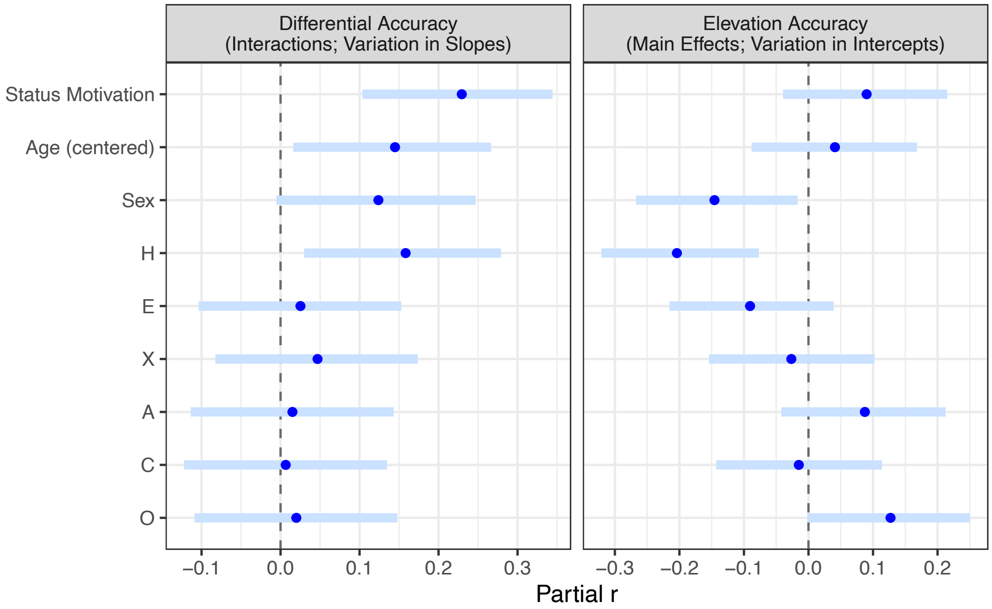

Figure 1. Associations between individual difference characteristics and indices of accuracy. The partial correlation estimates and 95% confidence intervals were computed from the model-estimated *t*-statistics and degrees of freedom. Note that differential accuracy associations are based on the interactions between the individual difference characteristics and peer-assessed status impacts, and the elevation accuracy associations are based on the main effects. H = Honesty-Humility; E = Emotionality; X = eXtraversion; A = Agreeableness; C = Conscientiousness; O = Openness. Status Motivation and the HEXACO traits are grand-mean centered and standardized, Female is an effect coded variable where -1 = Male, 1 = Female. Age is grand-mean centered but not standardized.

The small but statistically significant interaction between status motivations and peer-assessed status impacts ($p = .0004$), suggests that participants who scored higher on our measure of status motivations tended to have steeper slopes (i.e., better differential accuracy) than those who scored lower on status motivations. Although the main effect estimate for status motivations is positive, it was not statistically significant ($p = .172$), so we do not have strong support a relationship between status motivations and participant intercepts (i.e., elevation accuracy).

Differential accuracy (i.e., variation in participant slopes) was further predicted by age ($p = .028$) and Honesty-Humility ($p = .016$), such that older participants and those who scored higher on Honesty-

Humility tended to have slightly better differential accuracy. We did not find evidence of reliable associations between differential accuracy and participant sex (*p* = .060) or other HEXACO traits (*p*s > .478), and the point estimates for the later are centered around zero.

Elevation accuracy (i.e., variation in participant intercepts) was statistically significantly associated with participant sex (*p* = .027) and Honesty-Humility (*p* = .002), but not other individual difference characteristics (*p*s > .053). Given that a participant with perfect elevation accuracy would have an intercept of zero, interpretation of the statistically significant elevation associations is aided by Figure 2, where the model-estimated participant intercepts are plotted as a function of sex and Honesty-Humility. Male participants tended to over-estimate status impacts in relation to their peers, while female participants tended to under-estimate (Figure 2A). The negative association between participant intercepts and Honesty-Humility appears to be driven by low-scoring participants tending to over-estimate status-impacts, high-scorers tending to under-estimate, and participants scoring closer to average levels of Honesty-Humility tending to be relatively more accurate (Figure 2B).

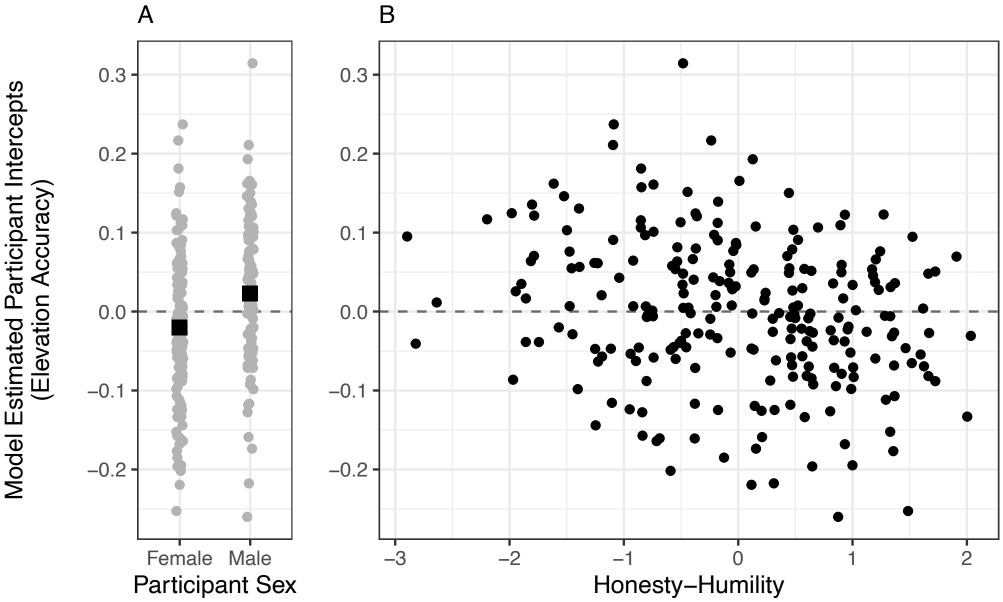

Figure 2. Depictions of participant intercepts (i.e., elevation accuracy) as a function of self-reported sex (Panel A), or scores on the Honesty-Humility scale (Panel B). Importantly, the statistical tests of these trends were based on associations with participants' latent intercepts, not the extracted estimates of their intercepts depicted here. The black squares on Panel B indicate the mean intercepts for each sex.

We note that participants' self-assessments of the status impacts of the 150 personal characteristics are very strongly positively associated with peer-assessments of the status impacts of those personal characteristics (*b* = .80, *r* = .99, *p* < .001). The estimated intercept was not statistically different from zero (*p* = .881), suggesting that the average person's status impact estimate will be zero when their peer's assessment is zero. The variance components reveal that there is generally little variation in slopes (i.e., differential accuracy; σ = .01) and intercepts (i.e., elevation accuracy; σ = .01) across participants. Together, these results suggest that agreement about status impacts between a given individual and their peers can be expected to be quite high. Additionally, the variance components reveal that latent slopes and intercepts are weakly negatively correlated (*r* = -.13), indicating that they are capturing largely distinct aspects of accuracy in the case of status-impact assessment as we have measured it.

To investigate whether there may be a sex difference in overall status motivations, we conducted a Welch two-sample *t*-test. The mean score of status motivation for men was 5.16 (*SD* = 1.10) and the mean for women was 5.27 (*SD* = .74). The difference between these means was not statistically significant, *t* = 0.91, *df* = 166.55, *p* = .367, *95% CI* [-.13, .36], *d* = .14. Thus, we cannot reject the hypothesis that the difference in status motivations between men and women is equal to zero.

Finally, Figure 3 shows all the correlations between the individual difference constructs we assessed in the current study. Of the 21 pairwise correlation between variables included in the current study, four were statistically significant after implementing a Holm correction for multiple tests. Status motivations were positively associated with Extraversion (*r* = .25, *p* < .001); Honesty-Humility was positively associated with both Conscientiousness (*r* = .24, *p* < .001) and Agreeableness (*r* = .27, *p* < .001); and Emotionality was negatively correlated with Extraversion (*r* = -.24, *p* < .001).

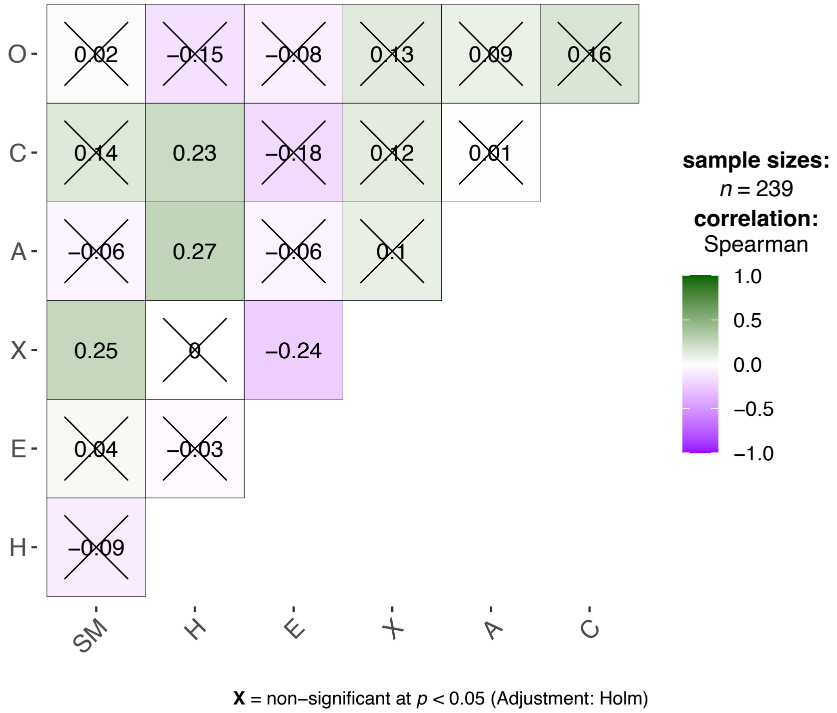

Figure 3. Correlation matrix depicting the magnitude, direction, and statistical significance of the correlations between the individual difference variables collected in the present study. Correlations with an "X" over them are not statistically significant. All *p*-values are adjusted for multiple tests using Holm's method. SM = Status Motivation; H = Honesty-Humility; E = Emotionality; X = eXtraversion; A = Agreeableness; C = Conscientiousness; O = Openness.

Registered Report Power Analysis

For the registered report, we based our target sample size on a bootstrap power analysis of our pilot data (c.f., Kleinman & Huang, 2016; Strong & Alvarez, 2019). While, we do not know the true effects, the bootstrapped power analysis provides insight into relationships between item and participant sample size settings and statistical power for the range of effect sizes that we observed in the pilot data, which are the best guess for expected effects in the replication samples. We iteratively bootstrapped 500 datasets based on different combinations of sample sizes for the items (ns = 20, 30, 40, 50) and participants (ns = 300, 400, 500, 600, 700, 800), which reflect different potential settings for the design of the proposed studies. We ran the main multilevel model with all focal interactions and controls on each bootstrapped data set and computed the mean effect size estimate and power (i.e., the percentage of statistically significant results) across the different setting combinations.

Figure S1 shows the relationships between power and the average effect size (partial *r* calculated from the *t*-statistics and degrees of freedom) across bootstrapped analyses for each sample size setting. The effect size results suggest that analyses based on 40 or 50 items tended to converge on similar effect estimates, whereas the estimated effects in analyses based on fewer items were comparably smaller (suggesting they are truncated in small samples of items); thus, we will present a random subset of 40 items to participants. Further, the power results suggest that we should have at least 80% power to detect effects larger than $r = .1$ with 40 items and between 400-800 participants. We also conducted a separate power analysis for the planned *t*-tests of status motivations using the *pwr.t.test* function in the *pwr* package (Champely et al., 2018), which suggested that we would need 393 participants per group (i.e., men and women) to be 80% powered to detect a small ($d = .2$) sex difference. Based on these two power analyses we set our target sample size at 800 participants (400 women and 400 women) per country.

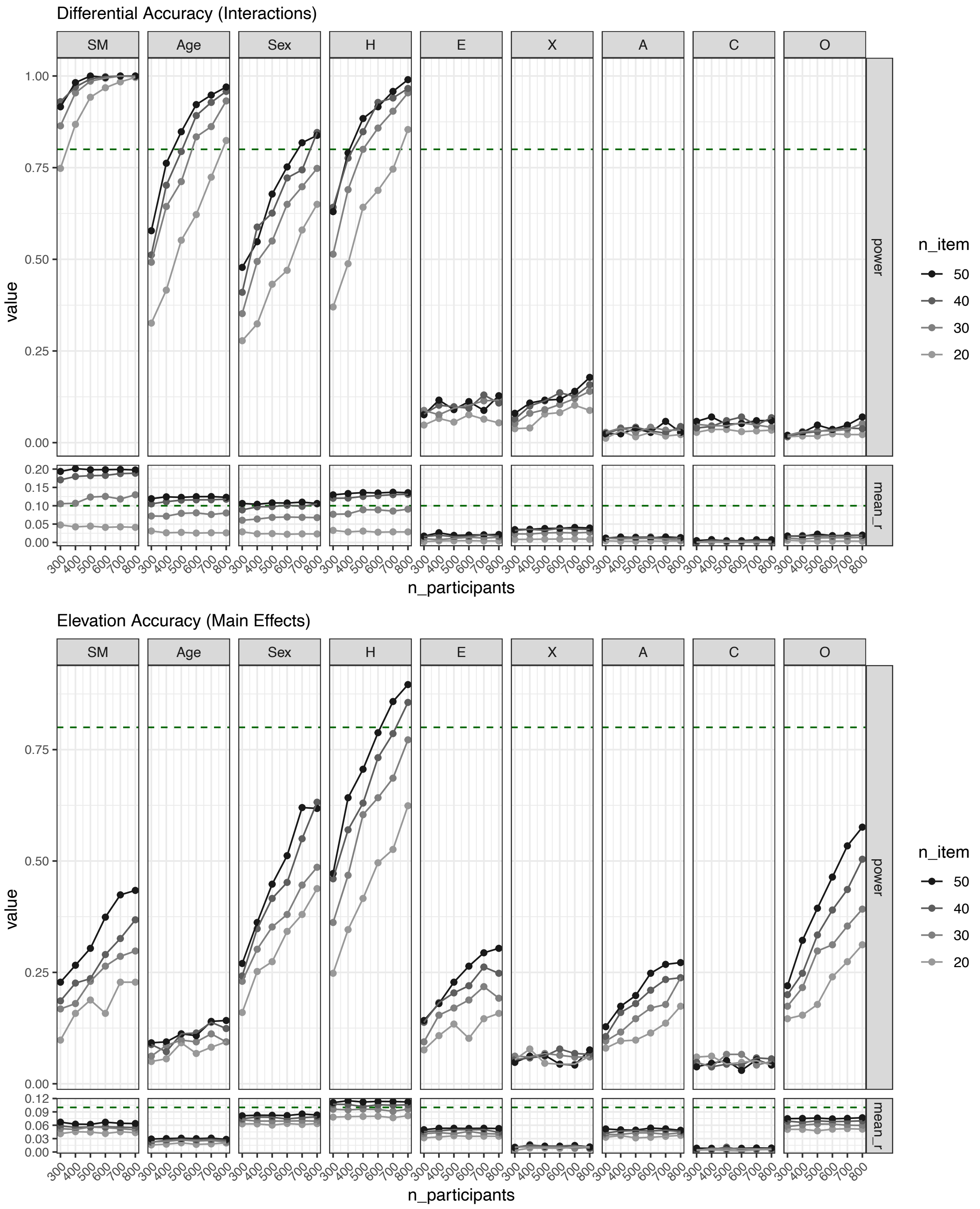

Figure S1. Results summary of bootstrapped power analyses for each combination of sample sizes for items and participants (n = 500 bootstrapped datasets per setting combination). The dashed lines depict the 80% power cutoff and the power plots (top panels) and demarcate where *r* = .1 on the mean effect size plots (bottom panels). SM = Status Motivation; H = Honesty-Humility; E = Emotionality; X = eXtraversion; A = Agreeableness; C = Conscientiousness; O = Openness. Status Motivations and the HEXACO traits are grand-mean centered and standardized, Participant sex is an effect coded variable (-1 = Male, 1 = Female). Age is grand-mean centered but not standardized.

Comparing the typical way of computing accuracy components to the multilevel model estimation of these components

To make sure that the parameters from the multilevel model correspond to appropriately to the indices of differential and elevation accuracy that are typically used, we looked at correlations between the indices derived from the two approaches. We computed differential accuracy as (a) the correlation between each person's self-assessed status impacts and their peer's assessments, and (b) the participants' random slope estimates extracted from the multilevel models where self-assessments and peer-assessments are standardized within-participants. We computed elevation accuracy as (a) the mean of the differences between each participant's self-assessments and the assessments of their peers for each item, and (b) the participants' random intercept estimates extracted from the multilevel models.

The minimum, maximum, mean and standard deviation of the accuracy indices are shown in the Table S1 below and the correlations among the different ways of computing accuracy are shown in the Figure S2. The strong correlations suggest in both countries that the multilevel models are correctly estimating the accuracy parameters that have been used in previous research. Note, however, that the MLM approach appropriately incorporates measurement error when estimating associations between the accuracy components and other individual difference constructs.

Table S1. *Ranges, Means, and Standard Deviations for the accuracy component estimates calculated the typical way (OG), and in the multilevel models (MLM).*

Accuracy Component	Min	Max	Mean	SD
differential_accuracy_MLM	-0.382	0.991	0.636	0.283
differential_accuracy_OG	-0.490	0.952	0.633	0.321
elevation_accuracy_MLM	-9.528	7.641	0.107	0.815
elevation_accuracy_OG	-3.318	2.177	0.001	0.548

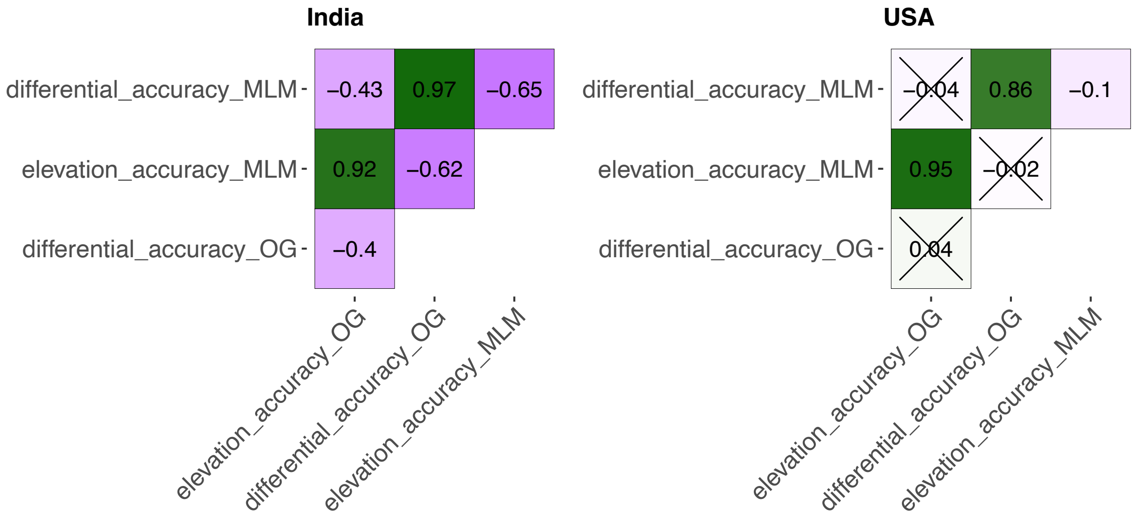

Figure S2. Ranges, Means, and Standard Deviations for the accuracy component estimates calculated the typical way (OG), and in the multilevel models (MLM).

Scale information

Table S2 shows the alpha reliabilities and other relevant information for the status motive and HEXACO scales. Reliability is high for the status motive measures, but generally low for the HEXACO measures. As noted in the main text, this low reliability for the HEXACO measure is to be expected given that each subscale is comprised of very few items and the items themselves are intended to cover the full breadth of the trait construct rather than a narrow piece of the trait space (de Vreis, 2013). Still, the reliability of some scales in India was especially poor (e.g., Agreeableness, Emotionality). However, the correlations between the individual differences were largely similar in each country, suggesting that they are tapping similar constructs in both countries even though the reliability is low. Still, caution is warranted in interpreting associations involving these measures, because differences in measurement error between the scales can drive differences in the results (Westfall & Yarkoni, 2016).

Table S2. *Scale information for status motive and HEXACO measures.*

COUNTRY	SCALE	STD.ALPHA	G6(SMC)	AVERAGE_R	MEAN	SD	MEDIAN_R
**INDIA**	Status_Motive	0.75935318	0.79380794	0.28286285	4.98544259	0.83881845	0.37517885
**INDIA**	Status_Motive2	0.71551743	0.70785198	0.29537386	4.99156429	0.97330774	0.34818793
**INDIA**	HonestyHumility	0.4318368	0.41885595	0.15967405	3.06728016	0.74445555	0.11998235
**INDIA**	Emotionality	0.07228973	0.13203322	0.01910844	2.89192229	0.58438642	-0.0279168
**INDIA**	Extraversion	0.14259328	0.29007183	0.03991727	3.4702454	0.6219184	-0.0091479
**INDIA**	Agreeableness	0.02236415	0.09464286	0.00568642	3.0396728	0.5736389	-0.0892616
**INDIA**	Conscientiousness	0.44466851	0.46047313	0.16679274	3.54969325	0.66501503	0.04864998
**INDIA**	Openness	0.21715745	0.24576498	0.06485162	3.83957055	0.56403398	0.12049352
**USA**	Status_Motive	0.88628169	0.88568173	0.4934676	4.3723236	1.13061638	0.48785496
**USA**	Status_Motive2	0.80824142	0.79334151	0.4126222	4.04142336	1.11450095	0.39871044
**USA**	HonestyHumility	0.58046858	0.54405274	0.25700436	3.98053528	0.77446973	0.30984226
**USA**	Emotionality	0.48296061	0.43342049	0.1893133	2.91575426	0.74186902	0.21508882
**USA**	Extraversion	0.66680149	0.63163719	0.33346819	3.59975669	0.84264024	0.28837468
**USA**	Agreeableness	0.3579179	0.30868198	0.12231298	2.9622871	0.64808431	0.13961552
**USA**	Conscientiousness	0.66817031	0.61764048	0.33484038	3.78315085	0.76335781	0.32157189
**USA**	Openness	0.56158912	0.4988835	0.24256272	3.83617194	0.70196256	0.21822023

Same results with another measure of status motivation

Figure S3 shows that the results of the focal model are essentially identical using an alternative measure—the Need for Status Scale developed by Flynn et al. (2006)—of status motivation that we included in the study to assess whether the results are dependent on the status motivation measure. As reported in the main text, there were no statistical differences in the results between the measures (see R code for full output tables).

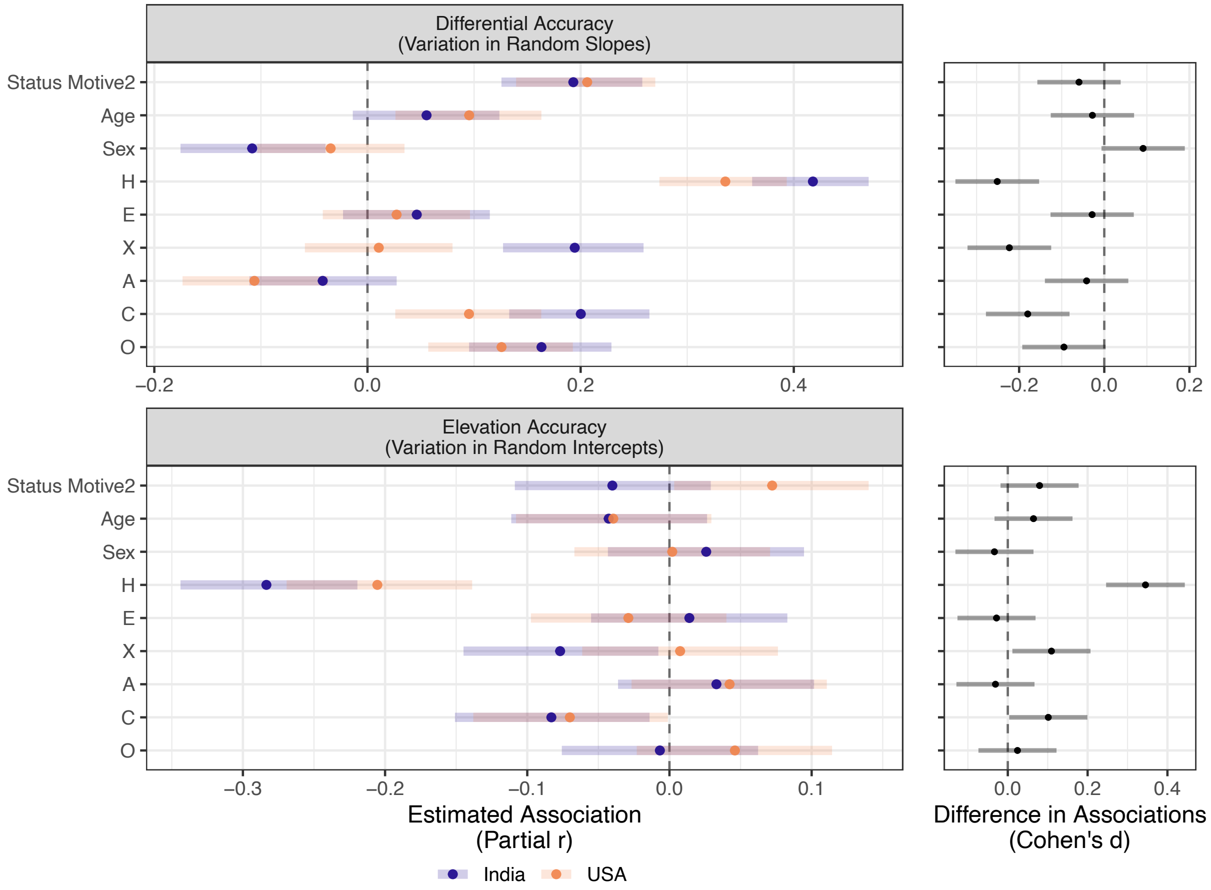

Figure S3. This figure reproduces the results of Figure 1 in the main text, using a different measure of status motivation in the analyses. Associations between individual difference characteristics and accuracy indices for participants in the USA (light orange) and India (dark purple). The plots on the left show model-estimated associations (converted to partial correlations) and 95% CI bands for the in each country. The plots on the right show exploratory contrast tests of the magnitude of the difference in the estimated associations between the two countries (converted to Cohen's d) and 95% CI for the difference. H = Honesty-Humility; E = Emotionality; X = eXtraversion; A = Agreeableness; C = Conscientiousness; O = Openness. Status Motivation and the HEXACO traits are grand-mean centered and standardized, Sex is an effect coded variable where -1 = Male, 1 = Female. Age is grand mean centered only.

Detailed model results tables

Table S3. *Associations between accuracy and two measures of status seeking without controls.*

	Model 1					Model 2				
Term	*Estimate*	*95% CI*	*t*	*p*	*df*	*Estimate*	*95% CI*	*t*	*p*	*df*
(Intercept)	0.21	0.16 – 0.27	7.37	**<0.001**	1627.59	0.21	0.16 – 0.27	7.35	**<0.001**	1626.58
scaled peer	0.52	0.50 – 0.55	50.02	**<0.001**	1623.88	0.53	0.50 – 0.55	50.03	**<0.001**	1622.50
country [USA]	-0.21	-0.29 – -0.13	-5.22	**<0.001**	1622.70	-0.21	-0.29 – -0.13	-5.20	**<0.001**	1621.69
status seeking	-0.00	-0.06 – 0.05	-0.16	0.876	1628.03					
scaled peer * country [USA]	0.22	0.19 – 0.25	14.93	**<0.001**	1624.18	0.22	0.19 – 0.25	14.91	**<0.001**	1622.82
scaled peer * status seeking	0.07	0.04 – 0.09	6.21	**<0.001**	1620.19					
country [USA] * status seeking	0.05	-0.04 – 0.13	1.10	0.271	1622.87					
(scaled peer * country [USA]) * status seeking	-0.04	-0.07 – -0.01	-2.95	**0.003**	1625.49					
status motive						-0.04	-0.10 – 0.01	-1.53	0.126	1626.75
scaled peer * status motive						0.07	0.05 – 0.09	6.40	**<0.001**	1623.38
country [USA] * status motive						0.09	0.01 – 0.17	2.16	**0.031**	1621.72
(scaled peer * country [USA]) * status motive						-0.06	-0.09 – -0.03	-4.23	**<0.001**	1626.96
**Random Effects**										
σ^2	0.51					0.51				
τ_{00}	0.67 $_{p\_id}$					0.66 $_{p\_id}$				
τ_{11}	0.08 $_{p\_id.scaled\_peer}$					0.08 $_{p\_id.scaled\_peer}$				
ρ_{01}	-0.50 $_{p\_id}$					-0.50 $_{p\_id}$				
ICC	0.58					0.58				
N	1630 $_{p\_id}$					1629 $_{p\_id}$				
Observations	65151					65118				
Marginal R^2 / Conditional R^2	0.274 / 0.694					0.274 / 0.694				

Table S4. *Associations between accuracy and two measures of status seeking with all controls.*

	Model 1					Model 2				
Term	*Estimate*	*95% CI*	*t*	*p*	*df*	*Estimate*	*95% CI*	*t*	*p*	*df*
(Intercept)	0.22	0.16 – 0.27	8.17	**<0.001**	1611.86	0.22	0.16 – 0.27	8.15	**<0.001**	1610.84
scaled peer	0.52	0.51 – 0.54	63.21	**<0.001**	1605.01	0.52	0.51 – 0.54	62.63	**<0.001**	1604.03
country [USA]	-0.21	-0.29 – -0.14	-5.77	**<0.001**	1606.03	-0.21	-0.29 – -0.14	-5.76	**<0.001**	1605.03
status seeking	-0.05	-0.11 – 0.01	-1.57	0.116	1611.50					
centered age	-0.01	-0.01 – 0.00	-1.67	0.095	1612.63	-0.01	-0.01 – 0.00	-1.74	0.082	1611.54
effect sex	0.03	-0.03 – 0.08	1.01	0.313	1611.95	0.03	-0.03 – 0.08	0.99	0.323	1610.94
HonestyHumility	-0.37	-0.43 – -0.31	-11.54	**<0.001**	1610.63	-0.37	-0.43 – -0.30	-11.86	**<0.001**	1609.89
Emotionality	0.02	-0.04 – 0.07	0.55	0.585	1611.12	0.02	-0.04 – 0.07	0.58	0.565	1610.24
Extraversion	-0.10	-0.16 – -0.03	-3.01	**0.003**	1612.07	-0.10	-0.16 – -0.03	-3.02	**0.003**	1611.07
Agreeableness	0.04	-0.02 – 0.09	1.29	0.197	1611.90	0.03	-0.02 – 0.09	1.23	0.218	1610.90
Conscientiousness	-0.11	-0.18 – -0.04	-3.25	**0.001**	1611.44	-0.11	-0.18 – -0.04	-3.28	**0.001**	1610.70
Openness	-0.01	-0.06 – 0.05	-0.26	0.793	1611.47	-0.01	-0.06 – 0.05	-0.23	0.817	1610.44
scaled peer * country [USA]	0.22	0.20 – 0.24	18.95	**<0.001**	1605.31	0.22	0.20 – 0.24	18.79	**<0.001**	1604.34
scaled peer * status seeking	0.06	0.04 – 0.08	6.44	**<0.001**	1594.30					
country [USA] * status seeking	0.07	-0.02 – 0.15	1.60	0.111	1605.94					
scaled peer * centered age	0.00	-0.00 – 0.00	1.83	0.068	1604.85	0.00	0.00 – 0.00	2.19	**0.029**	1604.11
country [USA] * centered age	0.01	-0.00 – 0.01	1.29	0.196	1609.76	0.01	-0.00 – 0.01	1.35	0.178	1608.68
scaled peer * effect sex	-0.03	-0.05 – -0.01	-3.57	**<0.001**	1605.18	-0.03	-0.04 – -0.01	-3.36	**0.001**	1604.23
country [USA] * effect sex	-0.03	-0.10 – 0.05	-0.67	0.503	1605.54	-0.03	-0.10 – 0.05	-0.66	0.511	1604.55
scaled peer * HonestyHumility	0.15	0.13 – 0.17	15.08	**<0.001**	1598.14	0.14	0.12 – 0.16	14.32	**<0.001**	1598.38
country [USA] * HonestyHumility	0.31	0.22 – 0.40	6.91	**<0.001**	1605.57	0.31	0.22 – 0.39	6.90	**<0.001**	1604.39
scaled peer * Emotionality	0.01	-0.00 – 0.03	1.52	0.128	1604.47	0.01	-0.00 – 0.03	1.61	0.107	1604.02
country [USA] * Emotionality	-0.02	-0.10 – 0.06	-0.57	0.571	1605.08	-0.02	-0.10 – 0.06	-0.54	0.586	1604.19
scaled peer * Extraversion	0.06	0.04 – 0.08	6.50	**<0.001**	1600.15	0.07	0.05 – 0.09	6.71	**<0.001**	1601.16
country [USA] * Extraversion	0.10	0.01 – 0.18	2.19	**0.028**	1606.14	0.10	0.01 – 0.19	2.25	**0.024**	1605.23
scaled peer * Agreeableness	-0.01	-0.03 – 0.00	-1.38	0.169	1595.84	-0.01	-0.03 – 0.01	-1.34	0.180	1595.69
country [USA] * Agreeableness	-0.02	-0.10 – 0.05	-0.62	0.536	1605.87	-0.02	-0.10 – 0.05	-0.55	0.582	1604.93
scaled peer * Conscientiousness	0.07	0.05 – 0.09	6.69	**<0.001**	1597.47	0.08	0.06 – 0.10	7.15	**<0.001**	1595.90

country [USA] * Conscientiousness	0.09	0.00 – 0.18	2.03	**0.042**	1606.53	0.09	0.00 – 0.18	2.02	**0.044**	1605.62
scaled peer * Openness	0.05	0.03 – 0.07	5.42	**<0.001**	1603.87	0.05	0.03 – 0.07	5.70	**<0.001**	1605.18
country [USA] * Openness	0.02	-0.06 – 0.10	0.49	0.627	1605.96	0.02	-0.06 – 0.10	0.49	0.624	1604.97
(scaled peer * country [USA]) * status seeking	-0.02	-0.04 – 0.01	-1.19	0.235	1605.07					
(scaled peer * country [USA]) * centered age	-0.00	-0.00 – 0.00	-0.56	0.577	1604.46	-0.00	-0.00 – 0.00	-0.94	0.347	1603.63
(scaled peer * country [USA]) * effect sex	0.02	-0.00 – 0.05	1.83	0.067	1606.61	0.02	-0.00 – 0.05	1.68	0.093	1605.74
(scaled peer * country [USA]) * HonestyHumility	-0.07	-0.10 – -0.04	-5.03	**<0.001**	1601.33	-0.06	-0.09 – -0.03	-4.29	**<0.001**	1600.51
(scaled peer * country [USA]) * Emotionality	-0.01	-0.03 – 0.02	-0.57	0.568	1606.36	-0.00	-0.03 – 0.02	-0.26	0.793	1605.71
(scaled peer * country [USA]) * Extraversion	-0.06	-0.09 – -0.03	-4.46	**<0.001**	1600.75	-0.06	-0.09 – -0.03	-4.22	**<0.001**	1599.82
(scaled peer * country [USA]) * Agreeableness	-0.01	-0.03 – 0.01	-0.83	0.406	1601.48	-0.01	-0.03 – 0.02	-0.54	0.586	1600.92
(scaled peer * country [USA]) * Conscientiousness	-0.05	-0.08 – -0.02	-3.60	**<0.001**	1599.25	-0.06	-0.09 – -0.03	-4.02	**<0.001**	1598.03
(scaled peer * country [USA]) * Openness	-0.02	-0.05 – 0.00	-1.90	0.058	1604.49	-0.02	-0.05 – 0.00	-1.92	0.055	1604.69
status motive						-0.05	-0.11 – 0.01	-1.77	0.078	1610.59
scaled peer * status motive						0.04	0.03 – 0.06	4.83	**<0.001**	1607.60
country [USA] * status motive						0.07	-0.01 – 0.15	1.72	0.085	1604.80
(scaled peer * country [USA]) * status motive						-0.01	-0.04 – 0.01	-0.91	0.365	1610.46
**Random Effects**										
σ^2	0.51					0.51				
τ_{00}	0.55 p_id					0.55 p_id				
τ_{11}	0.04 p_id.scaled_peer					0.04 p_id.scaled_peer				
ρ_{01}	-0.35 p_id					-0.35 p_id				
ICC	0.53					0.53				
N	1630 p_id					1629 p_id				
Observations	65151					65118				
Marginal R^2 / Conditional R^2	0.345 / 0.692					0.345 / 0.692				

Supplemental References

Champely, S., Ekstrom, C., Dalgaard, P., Gill, J., Weibelzahl, S., Anandkumar, A., ... & De Rosario, M. H. (2018). Package ‘pwr’. *R package version*, *1*(2).

De Vries, R. E. (2013). The 24-item brief HEXACO inventory (BHI). *Journal of Research in Personality*, *47*(6), 871-880.

Flynn, F. J., Reagans, R. E., Amanatullah, E. T., & Ames, D. R. (2006). Helping one's way to the top: self-monitors achieve status by helping others and knowing who helps whom. *Journal of personality and social psychology*, *91*(6), 1123.

Kleinman, K., & Huang, S. S. (2016). Calculating power by bootstrap, with an application to cluster-randomized trials. *EGEMs*, *4*(1).

Strong, R. W., & Alvarez, G. (2019, November 13). Using simulation and resampling to improve the statistical power and reproducibility of psychological research. https://doi.org/10.31234/osf.io/2bt6q

Westfall, J., & Yarkoni, T. (2016). Statistically controlling for confounding constructs is harder than you think. *PloS one*, *11*(3), e0152719.
